# Comparative Study on Biochemical Properties and Antioxidative Activity of Cuttlefish (*Sepia officinalis*) Protein Hydrolysates Produced by Alcalase and *Bacillus licheniformis* NH1 Proteases

**DOI:** 10.4061/2011/107179

**Published:** 2011-10-03

**Authors:** Rafik Balti, Ali Bougatef, Nedra El Hadj Ali, Naourez Ktari, Kemel Jellouli, Naima Nedjar-Arroume, Pascal Dhulster, Moncef Nasri

**Affiliations:** ^1^Laboratoire de Génie Enzymatique et de Microbiologie Ecole Nationale d'Ingénieurs de Sfax, Université de Sfax, B P 1173, Sfax 3038, Tunisia; ^2^Laboratoire de Procédés Biologiques, Génie Enzymatique et Microbien, IUT A Lille I, BP 179, 59653 Villeneuve d'Ascq Cedex, France

## Abstract

Antioxidative activities and biochemical properties of protein hydrolysates prepared from cuttlefish (*Sepia officinalis*) using Alcalase 2.4 L and *Bacillus licheniformis* NH1 proteases with different degrees of hydrolysis (DH) were determined. For the biochemical properties, hydrolysis by both enzymes increased protein solubility to above 75% over a wide pH range. The antioxidant activities of cuttlefish protein hydrolysates (CPHs) increase with increasing DH. In addition, all CPHs exhibited antioxidative activity in a concentration-dependent manner. NH1-CPHs generally showed greater antioxidative activity than Alcalase protein hydrolysates (*P* < 0.05) as indicated by the higher 1,1-diphenyl-1-picryhydrazyl (DPPH) radical scavenging activity and ferrous chelating activity. Both Alcalase and NH1 protein hydrolysates were able to retard lipid peroxidation and *β*-carotene-linoleic acid oxidation. Alcalase-CPH (DH = 12.5%) and NH1-CPH (DH = 15%) contained 75.36% and 80.11% protein, respectively, with histidine and arginine as the major amino acids, followed by glutamic acid/glutamine, serine, lysine, and leucine. In addition, CPHs have a high percentage of essential amino acids made up 48.85% and 50.04%. Cuttlefish muscle protein hydrolysates had a high nutritional value and could be used as supplement to poorly balanced dietary proteins.

## 1. Introduction

Free radical-mediated lipid peroxidation and antioxidants are attracting considerable research interest in many areas. Lipid oxidation is one of the major deteriorative processes in many types of foods, leading to the changes in food quality and nutritional value. Additionally, potentially toxic reaction products can be produced [1]. In particular, investigators report that free radicals, generated by oxidation, play a critical role in a variety of health disorders, including the processes of ageing, cancer, diabetes mellitus, inflammation, coronary heart, and neurological disorders, such as Alzheimer's disease [2]. Therefore, it is important to inhibit the oxidation and formation of free radicals occurring in the living body and foodstuffs [3]. 

Some synthetic antioxidative agents, such as butylated hydroxyanisole (BHA), butylated hydroxytoluene (BHT), and propyl gallate, are commonly used as free radical scavengers in food and biological systems. Although, these synthetic antioxidants show stronger antioxidant activity than those of natural antioxidants such as *α*-tocopherol and ascorbic acid, the use of these chemical compounds has begun to be restricted because of their induction of DNA damage and their toxicity [4]. Thus, increasing attention has been directed to the development of safe and effective functional foods and antioxidative agents from natural sources, especially peptides derived from hydrolyzed food proteins.

Recently, protein hydrolysates from several fish species, such as silver carp (*Hypophthalmichthys molitrix*) [5], brownstripe red snapper (*Lutjanus vitta*) [6], sardinelle (*Sardinella aurita*) [7], smooth hound (*Mustelus mustelus*) [8], oyster (*Crassostrea gigas*) [9], yellow stripe trevally (*Selaroides leptolepis*) [10], round scad (*Decapterus maruadsi*) [11], yellowfin sole (*Limanda aspera*) [12], herring (*Clupea harengus*) [13], and mackerel (*Scomber austraasicus*) [14], have been reported to possess antioxidative activities. 

The operational conditions employed in the processing of protein isolates, the type of protease, and the degree of hydrolysis affect the antioxidant activity [15]. Especially the, proteinases used can affect both the functional properties and antioxidative activity of the protein hydrolysate obtained [12]. Protein hydrolysate from Alaska pollack frame prepared by mackerel intestine crude enzyme exhibited antioxidative activity in a linoleic acid model system [16]. Prawn hydrolysate prepared using pepsin showed the most potent antioxidative activity than those prepared by other enzymes [17]. Levels and compositions of free amino acids and peptides were reported to determine the antioxidant activities of protein hydrolysates [14]. Moreover, the utilization of proteins or their hydrolysates for food and/or cosmetic applications not only presents additional advantages over other antioxidants but also confers nutritional and functional properties [18].

Recently, protein hydrolysates from cuttlefish (*Sepia officinalis*) enriched in angiotensin I-converting enzyme inhibitory peptides have been produced successfully using Alcalase and *Bacillus licheniformis *NH1 proteases [19]. Nevertheless, a little information regarding the characteristic and antioxidative activity of hydrolysates prepared using both enzymes has been reported. The objective of this study was to investigate the antioxidative activity of protein hydrolysates from cuttlefish muscle prepared using Alcalase and *Bacillus licheniformis *NH1 proteases in a model system and the effect of concentration on their activities. Meanwhile, solubility of hydrolysates derived from cuttlefish muscle was evaluated.

## 2. Materials and Methods

### 2.1. Reagents

1,1-diphenyl-2-picrylhydrazyl (DPPH), 3-(2-pyridyl)-5,6-bis(4-phenyl-sulphonic acid)-1,2,4-triazine (Ferrozine), butylated hydroxyanisole (BHA), *α*-tocopherol, and linoleic acid were purchased from Sigma-Aldrich, Inc. (St. Louis, Mo, USA). All other chemicals, namely ammonium thiocyanate, ferric chloride, EDTA, Tween-40, and sodium hydroxide were of analytical grade.

### 2.2. Fish Sample

Cuttlefish (*S. officinalis*), in the size range of 8–10 cuttlefish/kg, was purchased from the fish market of Sfax city, Tunisia. The samples were packed in polyethylene bags, placed in ice with a sample/ice ratio of approximately 1 : 3 (w/w), and transported to the research laboratory within 30 min. The mantle was cleaned, deskinned, and eviscerated and then stored in sealed plastic bags at −80°C until used.

### 2.3. Enzyme

The crude enzyme preparation from *B. licheniformis* NH1 [20] and Alcalase 2.4 L obtained from Novo Nordisk (Bagsverd, Denmark) was used for the production of protein hydrolysates. Protease activity was determined according to the method of Kembhavi et al. [21] using casein as a substrate. One unit of protease activity was defined as the amount of enzyme required to liberate 1 *μ*g tyrosine per minute under the experimental conditions used.

### 2.4. Production of Protein Hydrolysates from Cuttlefish Muscle

Cuttlefish (*S. officinalis*) muscle (500 g), in 1000 mL distilled water, was first minced using a grinder (Moulinex Charlotte HV3, France) and then cooked at 90°C for 20 min to inactivate endogenous enzymes. The cooked muscle sample was then homogenized in a Moulinex blender for about 2 min and hydrolyzed with enzymes under optimal conditions: the crude enzyme preparation from *B. licheniformis* NH1 (pH 10.0 and 50°C) and Alcalase 2.4 L (pH 8.0 and 50°C). The enzyme was added to the reaction at the same enzyme/substrate ratio (*E/S* = 3 U/mg) to compare hydrolytic efficiencies. During the reaction, the pH of the mixture was maintained constant by continuous addition of 4 M NaOH solution. After the required digestion time, the reaction was stopped by heating the solution at 80°C during 20 min to inactivate the enzyme. The cuttlefish muscle protein hydrolysates were then centrifuged at 5000 ×g for 20 min to separate insoluble and soluble fractions. Finally, the soluble phase was freeze dried using freeze dryer (Bioblock Scientific Christ ALPHA 1-2, IllKrich-Cedex, France) and stored at −20°C for further use.

### 2.5. Degree of Hydrolysis Determination (DH)

The degree of hydrolysis (DH), defined as the percent ratio of the number of peptide bonds broken (*h*) to the total number of peptide bonds in the studied substrate (*h*_tot_), was calculated from the amount of base (NaOH) added to keep the pH constant during the hydrolysis [22] as given below:

(1) $$\text{DH}\;\left(\%\right)=\frac{h}{h_{\text{tot}}}\times 100=\frac{B\times Nb}{MP}\times \frac{1}{\alpha }\times \frac{1}{h_{\text{tot}}}\times 100,$$

where *B* is the amount of NaOH consumed (mL) to keep the pH constant during the reaction, Nb is the normality of the base, MP is the mass (g) of protein (N × 6.25), and **α** is the average degree of dissociation of the *α*-NH_2_ groups released during hydrolysis expressed as:

(2) $$\alpha =\frac{10^{pH-pK}}{{1+10}^{pH-pK}},$$

where pH and pK are the values at which the proteolysis was conducted. The total number of peptide bonds (*h*_tot_) in a fish protein concentrate was assumed to be 8.6 meq/g [22].

### 2.6. Proximate Analysis

Moisture and ash content were determined according to the AOAC [23] standard methods 930.15 and 942.05, respectively. Total nitrogen content of the substrate and selected hydrolysate products was determined by using the Kjeldahl method. Crude protein was estimated by multiplying total nitrogen content by the factor of 6.25. Lipids were determined gravimetrically after Soxhlet extraction of dried samples with hexane. All measurements were performed in triplicate. The protein and fat contents were expressed on a dry weight basis.

### 2.7. Amino Acid Analysis

For analysis of amino acids, the dry samples were dissolved in distilled water at 1 mg/mL, and 50 *μ*L of each sample were dried and hydrolysed in vacuum-sealed glass tube at 110°C for 24 h in the presence of constant boiling 6 N HCl containing 1% (w/v) phenol and using norleucine as internal standard. After hydrolysis, samples were again vacuum dried, dissolved in application buffer and injected into a Beckman 6300 amino acid analyzer (Beckman Instruments Inc., Fullerton, Calif, USA).

### 2.8. Solubility

Solubility of cuttlefish protein hydrolysates was carried out according to Tsumura et al. [24] with slight modifications. Briefly, 200 mg of freeze-dried hydrolysates of cuttlefish protein were suspended in 20 mL deionized distilled water, and the pH of the mixture was adjusted to different values from 2.0 to 11.0 using either 2 N HCl or 2 N NaOH solutions. The mixtures were stirred for 10 min at room temperature (25 ± 1°C) and then centrifuged at 8000 ×g for 10 min. After appropriate dilution, the nitrogen content in the supernatant was determined by Biuret method [25]. The nitrogen solubility of the sample, defined as the amount of soluble nitrogen from the total nitrogen, was calculated as follows:

(3) $$\begin{array}{cc} & \text{Nitrogen solubility (\%)}\\ & \;\;=\frac{\text{Supernatant nitrogen concentration}}{\text{Sample nitrogen concentration}}\times 100. \end{array}$$

Solubility analysis was carried out in triplicate.

### 2.9. Determination of Antioxidant Activities

#### 2.9.1. Inhibition of Linoleic Acid Autoxidation

Inhibition activity of *in vitro* lipid peroxidation of cuttlefish protein hydrolysates was determined by assessing their ability to inhibit oxidation of linoleic acid in an emulsified model system [26]. Briefly, freeze-dried hydrolysates of cuttlefish protein (5 mg) were dissolved in 2.5 mL of 50 mM phosphate buffer (pH 7.0) and added to a 2.5 mL of 50 mM linoleic acid in ethanol (95%). The final volume was then adjusted to 6.25 mL with distilled water. The mixture was incubated in a 10 mL tube with silicon rubber caps at 45°C for 12 days in a dark, and the degree of oxidation was evaluated by measuring the ferric thiocyanate values according to the method of Mitsuda et al. [27]. Aliquot (0.1 mL) of reaction mixture was mixed with 4.7 mL of 75% ethanol followed by the addition of 0.1 mL of 30% ammonium thiocyanate and 0.1 mL of 20 mM ferrous chloride solution in 3.5% HCl. After stirring for 3 min, the degree of colour development, which represents the linoleic acid oxidation, was measured at 500 nm. The antioxidative capacity of the inhibition of peroxide formation in linoleic acid system was expressed as follows:

(4) $$\text{Inhibition (\%) }=\left[1-\frac{A_{\text{500}}\;\;\text{of sample}}{A_{\text{500}}\;\;\text{of control}}\right]\times 100,$$

*α*-tocopherol, a natural antioxidant agent, and BHA, a synthetic antioxidant agent, were used as reference, and distilled water as control. 

#### 2.9.2. 1,1-Diphenyl-2-Picrylhydrazyl (DPPH) Radical Scavenging Activity

DPPH radical-scavenging activity was measured, using the method described by Bersuder et al. [28]. A 500 *μ*L test sample was mixed with 500 *μ*L of 99.5% ethanol and 125 *μ*L of 99.5% ethanol containing 0.02% DPPH. This mixture was shaken then kept in a dark at room temperature for 60 min before measuring absorbance at 517 nm. DPPH radical-scavenging activity was calculated according to the following equation:

(5) $$\begin{array}{cc} & \text{DPPH radical-scavenging activity (\%)}\\ & \;\;=\;\left[1-\frac{A_{\text{517}}\;\;\text{of sample}}{A_{\text{517}}\;\;\text{of control}}\right]\times 100. \end{array}$$

The control was conducted in the same manner except that distilled water was used instead of sample. A lower absorbance of the reaction mixture indicated a higher DPPH scavenging activity. Butylated hydroxyanisole (BHA) was used as a standard.

#### 2.9.3. Ferrous Chelating Activity

The chelating activity on Fe^2+^ was determined, using the method of Decker and Welch [29]. One millilitre of sample solution was mixed with 3.7 mL of distilled water. The mixture was then reacted with 0.1 mL of 2 mM FeCl_2_ and 0.2 mL of 5 mM 3-(2-pyridyl)-5,6-bis(4-phenyl-sulfonic acid)-1,2,4-triazine (ferrozine) for 20 min at room temperature. The absorbance was read at 562 nm. The control was prepared in the same manner except that distilled water was used instead of the sample. EDTA was used as reference. Chelating activity (%) was then calculated as follows [29]:

(6) $$\text{Chelating activity (\%) }=\;\left[1-\frac{A_{\text{562}}\;\;\text{of sample}}{A_{\text{562}}\;\;\text{of control}}\right]\times 100.$$

#### 2.9.4. *β*-Carotene-Linoleic Acid Assay

Antiautooxidant activity was assayed using the *β*-carotene bleaching method [30]. In brief, 0.5 mg *β*-carotene in 1 mL chloroform was mixed with 25 *μ*L of linoleic acid and 200 *μ*L of Tween-40. The chloroform was evaporated under vacuum at 45°C; then 100 mL distilled water was added, and the resulting mixture was vigorously stirred. The emulsion obtained was freshly prepared before each experiment. An aliquot (2.5 mL) of the *β*-carotene-linoleic acid emulsion was transferred to tubes containing 0.5 mL of each sample. The tubes were immediately placed in water bath and incubated at 50°C for 2 h. Thereafter, the absorbance of each sample was measured at 470 nm. A control consisted of 0.5 mL of distilled water instead of the sample solution. BHA was used as positive standard:

(7) $$\begin{array}{cc} & \text{Antioxidant activity (\%)}\\ & \;\;=\left[1-\frac{\left({\text{Abs}}_{\text{sample}}^{0}-{\text{Abs}}_{\text{sample}}^{\text{120}}\right)}{\left({\text{Abs}}_{\text{control}}^{\text{0}}-{\text{Abs}}_{\text{control}}^{\text{120}}\right)}\right]\times 100. \end{array}$$

### 2.10. Statistical Analysis

One-way analysis of variance (ANOVA) was used, and mean comparison was performed by Duncan's multiple range test [31]. Statistical analyses were performed with Statgraphics ver. 5.1, professional edition (Manugistics Corp., Rockville, MD, USA). Differences were considered significant at *P* < 0.05.

## 3. Results and Discussion

### 3.1. Preparation of Protein Hydrolysates from Cuttlefish Muscle

It has been demonstrated that biological activities of proteins can be increased through hydrolysis with certain enzymes, and some peptides or fractions possess stronger activity than others [19]. Furthermore, the specificity of the enzyme used for the proteolysis, the conditions used during hydrolysis and the DH greatly influenced the molecular weight and amino acid composition of protein hydrolysates, and thus their biological activities [32]. 

**Figure 1:**
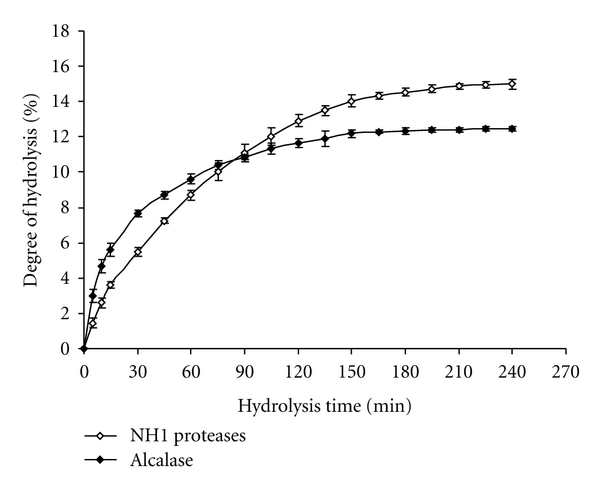
Degree of hydrolysis (DH) of CPHs during hydrolysis with Alcalase and NH1 proteases at 3 U/mg enzyme/substrate. Bars represent standard deviations from triplicate determinations.

The hydrolysis of the cuttlefish proteins with NH1 proteases or Alcalase was characterized by a high rate of hydrolysis for the first 1 h (Figure 1). The rate of enzymatic hydrolysis was subsequently decreased, and then the enzymatic reaction reached the steady-state phase when no apparent hydrolysis took place. The shape of hydrolysis curves is similar to those previously published for many protein substrates such as fish [33], whey [34], and wheat gluten [35]. The decrease in the reaction rate could be explained by a decrease in the concentration of peptide bonds available for hydrolysis, enzyme deactivation, and/or the inhibition of the enzyme by the products formed at high degree of hydrolysis. These products act as effective substrate competitors to the undigested or partially digested fish proteins.

With the same *E/S* ratio, NH1 proteases showed higher DH values for cuttlefish protein hydrolysis than Alcalase beyond 90 min hydrolysis period. The higher (*P* < 0.05) level of DH by NH1 proteases treatment may be due to the fact that NH1 crude enzyme contains multiple proteases and, therefore, is a more efficient enzyme choice than Alcalase for preparing cuttlefish protein hydrolysates. Therefore, the susceptibility, to hydrolysis, of cuttlefish muscle proteins depends on the type of enzyme used.

### 3.2. Proximate Composition

Proximate composition of soluble fractions of freeze-dried CPHs compared to that of undigested cuttlefish proteins is shown in Table 1. The proximate composition of the undigested cuttlefish muscle proteins (UCMP) showed that it had high protein content (79.15 ± 0.48%). The ash and lipid contents of UCMP were 6.08 ± 0.71% and 5.19 ± 0.24%, respectively. Alcalase and NH1 CPHs powders had a white to light yellow color appearance with almost no fishy odor and taste.

The protein content of CPHs varied with both enzyme treatments (Table 1). After 4 h of hydrolysis, Alcalase-CPH had the least protein content (75.36 ± 0.68%). However, NH1-CPH showed a higher protein content (80.11 ± 0.75%). During hydrolysis, proteins were solubilised, and the insoluble nonprotein matter was removed, resulting in the high protein content in the resulting hydrolysate [36]. The obtained results are similar to those of other studies on fish protein hydrolysates that have ranged protein from 63.4 to 90.8% [13, 37]. Generally, alkaline proteases exhibit a greater capability to solubilize fish protein compared to neutral and acidic proteases, with exception of pepsin [38]. 

Interestingly, both dried cuttlefish protein hydrolysates had low lipid content values 0.68 ± 0.02% and 0.91 ± 0.05%, in Alcalase-CPH and NH1-CPH, respectively. A low lipid contents were reported in salmon [39] and herring [40] protein hydrolysates. The retention of a high amount of fat in the final products may limit the use of this ingredient in food applications, because fish protein hydrolysates with high lipid content can have an undesirable taste and darken owing to changes in the lipids [41]. According to Spinelli et al. [42], the level of lipid residues in fish protein hydrolysates must be low.

The ash content was 10.12% and 12.44% in Alcalase-CPH, and NH1-CPH respectively, representing the salt formed during pH adjustment using alkaline solution (NaOH, 4 M). These results are similar to those of other published studies on fish protein hydrolysates [39, 43].

### 3.3. Solubility

**Figure 2:**
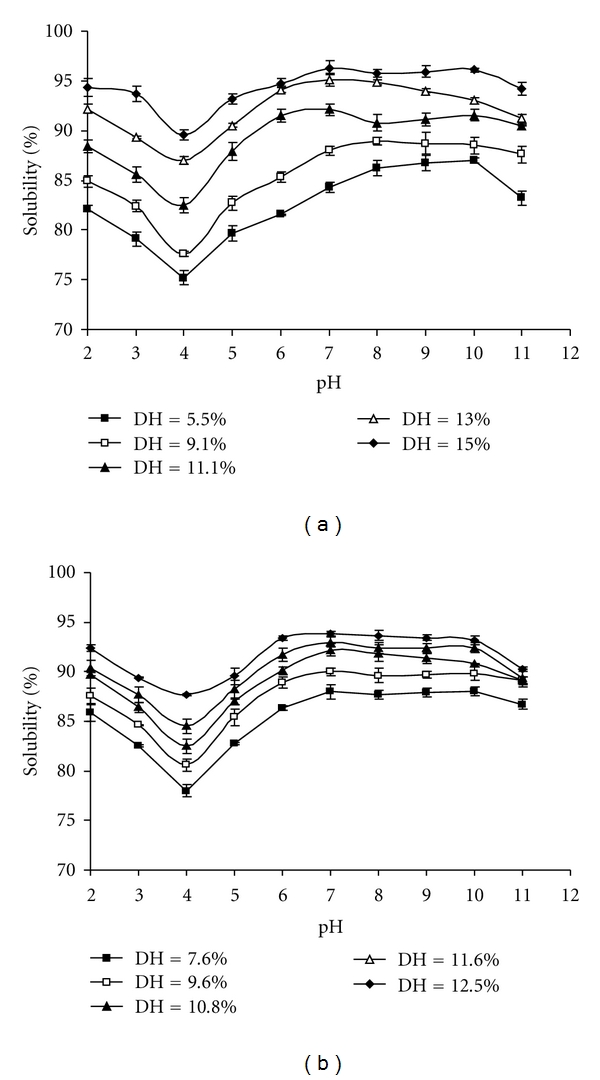
Solubility profiles of CPHs with different degrees of hydrolysis as influenced by pHs: (a) DH = 5.5%, DH = 9.1%, DH = 11.1%, DH = 13.0%, DH = 15.0% with proteases from *B. licheniformis* NH1, respectively; (b) DH = 7.6%, DH = 9.6%, DH = 10.8%, DH = 11.6%, DH = 12.5% with Alcalase, respectively. Bars represent standard deviations from triplicate determinations.

Functional properties influence the usefulness of an ingredient in food and govern the physical behavior during preparation, processing, and storage [44]. Solubility is one of the most important properties of proteins and protein hydrolysates [45]. Many other functional properties such as emulsification and foaming are affected by solubility. The pH solubility profiles of CPHs at different DH are shown in Figure 2. All CPHs presented typical bell-shaped solubility curves with minimum solubility at pH 4, which may correspond to the isoelectric point of protein hydrolysates, and high solubility at acidic and alkaline pH. The solubilities of CPHs were quite low at pH 4, whereas solubilities above 78% were noticeable at other pHs tested. Undigested cuttlefish muscle protein (UCMP) was less soluble than the hydrolysates, having a solubility of 7.86 ± 0.026% and 15.86 ± 0.056% at pH 4.0 and 9.0, respectively (data not shown). 

As shown in Figure 2, the solubility increased with increasing protein hydrolysis. At pH 7.0, the solubility of Alcalase-CPH (DH = 12.5%) and NH1-CPH (DH = 15%) reached about 93.8 ± 0.22 and 96.33 ± 0.7%, respectively, significantly higher (*P* < 0.05) than that of the UCMP (12.05 ± 0.017%). The obtained results are in line with those of Klompong et al. [10] and Gbogouri et al. [37] who reported that hydrolysates of yellow stripe trevally meat protein and salmon byproduct had an excellent solubility at high degrees of hydrolysis. From these results, we can deduce that the solubility increases with the protein fraction with lower molecular mass at higher degrees of hydrolysis. The smaller peptides are expected to have proportionally more polar residues, with the ability to form hydrogen bonds with water and increase solubility [37].

In addition, the lowest solubility of CPHs observed at pH 4.0 could be attributed to both net charge of peptides, which increase as pH moves away from pI, and surface hydrophobicity, that promotes the aggregation via hydrophobic interaction [46]. The pH affects the charge on the weakly acidic and basic side chain groups, and hydrolysates generally show low solubility at their isoelectric points [47].

### 3.4. Antioxidant Activity

#### 3.4.1. Inhibition of Linoleic Acid Autoxidation

*In vitro* lipid peroxidation inhibition activities of CPHs were determined by assessing their ability to inhibit oxidation of linoleic acid in an emulsified model system. As shown in Table 2, all hydrolysates with different DH could act as significant retarders (*P* < 0.05) of lipid peroxidation. The hydrolysates inhibiting lipid oxidation exhibited a nonlinear pattern. For the tow protease preparations used for hydrolysis reaction of cuttlefish muscle protein, the effect of inhibiting lipid oxidation increased initially and peaked on 4.0 h of hydrolysis. According to Dong et al. [48], the effect of inhibiting lipid oxidation of Alcalase-hydrolyzed carp protein increased initially and peaked on 1.5 h of hydrolysis, followed by a slight decline during the 6 h of hydrolysis. Wu et al. [14] found that the antioxidant activity of hydrolysates derived from mackerel protein reached a maximum after 10 h of hydrolysis and then declined slightly during the 25 h of hydrolysis. 

**Figure 3:**
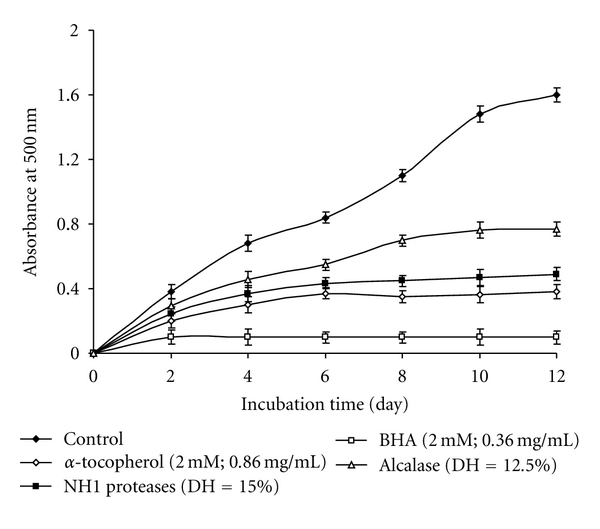
Comparison of inhibition of lipid peroxidation between NH1-CPH (DH = 15.0%), Alcalase-CPH (DH = 12.5%), at 1 mg/mL, *α*-tocopherol (2.0 mM; 0.86 mg/mL), and BHA (2.0 mM; 0.36 mg/mL). Bars represent standard deviations from triplicate determinations.

The comparative study between NH1-CPH (DH = 15%) and Alcalase-CPH (DH = 12.5%) and commercial antioxidants (*α*-tocopherol and BHA) on the inhibition of lipid peroxidation were conducted and illustrated in Figure 3. Only NH1-CPH presents a comparable effect than natural antioxidant *α*-tocopherol. However, both hydrolysates have moderate protective effect on lipid peroxidation in comparison with a synthetic antioxidant BHA.

Generally, the lack of a direct relationship between antioxidant activity and DH suggested that the specific composition (e.g., type of peptides, ratio of different freed amino acids) was an important factor as well [49]. Many researchers reported that low molecular weight peptides showed higher antioxidant activity [50]. In addition, Kong and Xiong [49] reported that if the hydrolysis of zein protein with Alcalase became too extensive (time of hydrolysis >4 h), the hydrolysate could reduce the peptide's ability to act as a physical barrier to prevent oxidants from reaching the lipid fraction in the liposome.

#### 3.4.2. DPPH Radical Scavenging Activity

DPPH is a stable free radical that shows maximum absorbance at 517 nm. When DPPH radicals encounter a proton-donating substrate such as an antioxidant, the radicals would be scavenged, and the absorbance is reduced [51]. The decrease in absorbance is taken as a measure for radical-scavenging. Thus, the DPPH radicals were widely used to investigate the scavenging activity of some natural compounds.  Table 3 shows the DPPH radical scavenging activities of CPHs with different DH. Both hydrolysates exhibited significant hydroxyl radical scavenging activity (*P* < 0.05).

The DPPH radical scavenging activity of CPHs increased with increasing DH. At all designated DH, NH1-CPH showed higher activity than did Alcalase-CPH (*P* < 0.05). For NH1 proteases, when DH was increased from 3.6% to 15%, the DPPH radical scavenging activity markedly increased from 25.0% to 71.5%. The result suggested that the peptides in different hydrolysates might be different in terms of chain length and amino acid sequence, which contributed to varying capabilities of scavenging DPPH radicals. The increase in DPPH radical scavenging activity of both protein hydrolysates was in agreement with Thiansilakul et al. [11] who reported the increase in DPPH radical scavenging activity as the DH of the hydrolysate from round scad muscle protein prepared using Flavourzyme and Alcalase increased. However, Klompong et al. [10] found that DPPH radical scavenging activity of protein hydrolysate prepared from the muscle of yellow stripe trevally using Flavourzyme and Alcalase decreased when DH increased. Invert correlation between DH and DPPH radical scavenging activity was obtained for protein hydrolysates prepared from alkaline-aided channel catfish protein isolates using Protamex [52]. You et al. [53] reported that loach protein hydrolysate showed the greater DPPH radical scavenging activity when DH increased. 

**Figure 4:**
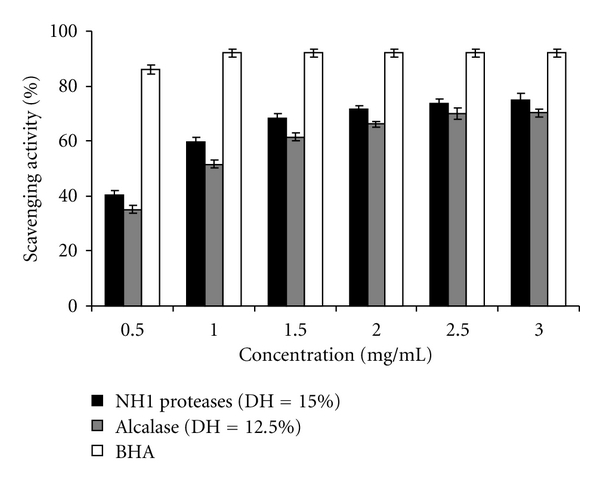
DPPH scavenging activity of NH1-CPH (DH = 15.0%) and Alcalase-CPH (DH = 12.5%) at different concentrations. BHA was used as positive control. Bars represent standard deviations from triplicate determinations.

The scavenging effect of all CPHs on DPPH radical scavenging was concentration dependent (Figure 4). The result clearly indicated that hydrolysate produced by NH proteases exhibited the highest radical scavenging activity (75.0 ± 2.26% at 3 mg/mL). However, the hydrolysate showed a lower radical-scavenging activity than BHA (89.87 ± 1.5%) at the same concentration.

The IC_50_ values were determined. The lower IC_50_ indicates higher free radical scavenging ability. Hydrolysate obtained by treatment with NH1 proteases showed an active radical scavenger with IC_50_ about 0.68 mg/mL ± 0.017 than Alcalase protein hydrolysate (IC_50_ = 0.99 mg/mL ± 0.024). DPPH is a stable free radical and can be scavenged with a proton-donating substance, such as an antioxidant [54]. Therefore, protein hydrolysates from cuttlefish muscle more likely contained peptides acting as hydrogen donors, thereby scavenging free radicals by converting them into more stable products.

#### 3.4.3. Ferrous Chelating Activity

Ferrous chelating activities of NH1-CPH and Alcalase-CPH, determined at a sample concentration of 2.0 mg/mL, are presented in Table 4. The chelating activity of hydrolysates increased with DH. NH1-CPH showed a higher chelating activity (*P* < 0.05) than Alcalase-CPH at any designated DH. Ferrous ion (Fe^2+^) is the most powerful prooxidant among metal ions [55], leading to the initiation and acceleration of lipid oxidation by interaction with hydrogen peroxide in a Fenton reaction to produce the reactive oxygen species, hydroxyl free radical (OH^•^) [56]. Therefore, chelation of metal ions by peptides in hydrolysates could retard the oxidative reaction. The result indicated that a higher DH rendered NH1-CPH and Alcalase-CPH with higher metal chelating activities. The shorter chain of peptides might lose their ability to form the complex with Fe^2+^. For both crude protease preparations used, NH1-CPH (DH = 15%) and Alcalase-CPH (DH = 12.5%), obtained after 4 h of hydrolysis, exhibited a higher ferrous-chelating abilities 65.0 ± 1.77% and 55.6 ± 1.45%, respectively. The increased metal chelating activity could be increased through hydrolysis with certain enzymes. 

Peptides in NH1-CPH and Alcalase-CPH could effectively chelate the Fe^2+^, leading to the retardation of initiation stage. The result indicated that the excessive hydrolysis of muscle protein resulted in the enhanced ferrous chelating activity, compared with the limited hydrolysis. The higher chelating activities of both hydrolysates were coincidental with the higher DPPH and *in vitro* lipid peroxidation inhibition activity, as the DH increased. Fe^2+^ chelating activity of round scad protein hydrolysate prepared using Alcalase showed the increase in chelating activity with increasing DH, but those treated with Flavourzyme showed no difference in activity at all DH tested [11]. With the same enzymes used, chelating activity of protein hydrolysate prepared from the muscle of yellow stripe trevally increased with increasing DH [10]. Higher ferrous chelating activity was reported for hydrolysate of silver carp using Alcalase and Flavourzyme when DH increased [48]. Apart from Fe, other transition metals, such as Cu and Co, can affect the rate of lipid oxidation and decomposition of hydroperoxide. Theodore et al. [52] reported that Cu^2+^ chelating activity of catfish protein hydrolysate increased with increasing DH.

Some proteins and peptides can chelate metal ions like Fe^2+^ due to the presence of carboxyl and amino groups in the side chains of acidic and basic amino acids [57]. Alcalase is endopeptidase capable of hydrolyzing proteins with broad specificity for peptide bonds and is prefered for the uncharged residue [10], whereas NH1 proteases is a mixture of multiple proteases, which can produce both amino acids and peptides [58]. Hydrolysates showing different antioxidative activities might be attributed to the differences in the exposed side chains of peptides as governed by the specificity of different proteases towards peptide bonds in the proteins [59]. DH also greatly influenced the peptide chain length. The higher DH was, the more cleavage of peptide chains took place. Peptides with various sizes and compositions had different capacities of scavenging or quenching free radicals [10, 11]. NH1 proteases and Alcalase more likely cleaved the peptide bonds in cuttlefish muscle at different positions, resulting in the different products with varying antioxidative activities. 

**Figure 5:**
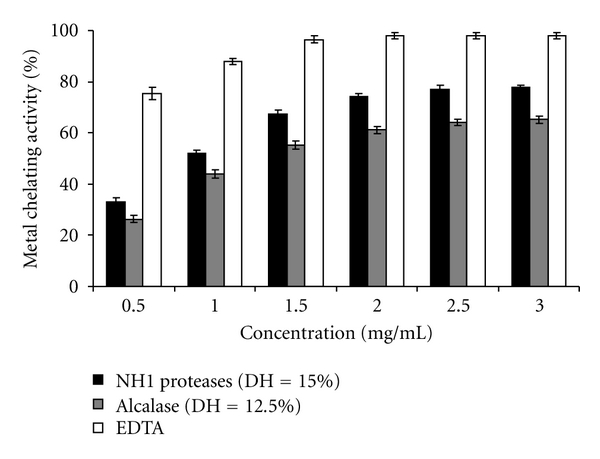
Relative chelating activity of NH1-CPH (DH =15.0%) and Alcalase-CPH (DH = 12.5%) at different concentrations. EDTA was used as positive control. Bars represent standard deviations from triplicate determinations.

The ferrous chelating activity, at different concentration, was also studied with NH1-CPH and Alcalase-CPH having, respectively, a DH of 15% and 12.5%, and compared with that of the EDTA (Figure 5). As shown in Figure 5, both activities increased with increasing hydrolysate concentration, and reached a maximum activity with 2 mg/mL, and further increase in hydrolysate concentration did not affect the activity. The IC_50_ values were about 0.94 ± 0.24 mg/mL and 1.25 ± 0.68 mg/mL, for NH1-CPH, and Alcalase-CPH respectively. However, both hydrolysates showed a lower metal chelating activity than did EDTA at all concentrations tested. For example, at 2 mg/mL the metal chelating activities of NH1-CPH, Alcalase-CPH, and EDTA were 74.0%  ± 1.55, 61.2%  ± 1.34, and 98%  ± 1.02, respectively.

#### 3.4.4. Antioxidant Activity in *β*-Carotene Linoleic Acid Emulsion Model System

The antioxidative activity of cuttlefish protein hydrolysates, NH1-CPH, and Alcalase-CPH were studied in *β*-carotene linoleic acid oxidation model system as presented in Table 5. When the oxidation of linoleic acid occurs, free radicals formed are able to attack the highly unsaturated *β*-carotene molecules. As a result, *β*-carotene is oxidized, leading to the losses in chromophore and characteristic orange colour of *β*-carotene [60]. The presence of antioxidant in linoleic acid emulsion system hinders *β*-carotene bleaching, due to the chain-breaking inhibition of lipid peroxidation by neutralizing the linoleic free radical formed. 

As shown in Table 5, NH1-CPH and Alcalase-CPH inhibited significantly (*P* < 0.05) the oxidation of *β*-carotene to different degrees. During the first hour of hydrolysis, Alcalase-CPH achieved rapidly a higher ability to prevent the bleaching of *β*-carotene (61.5 ± 1.66%) than NH1-CPH (56.5 ± 2.31%). When hydrolysis reached a final DH, both hydrolysates showed a similar antioxidant activity, approximately 70%.

**Figure 6:**
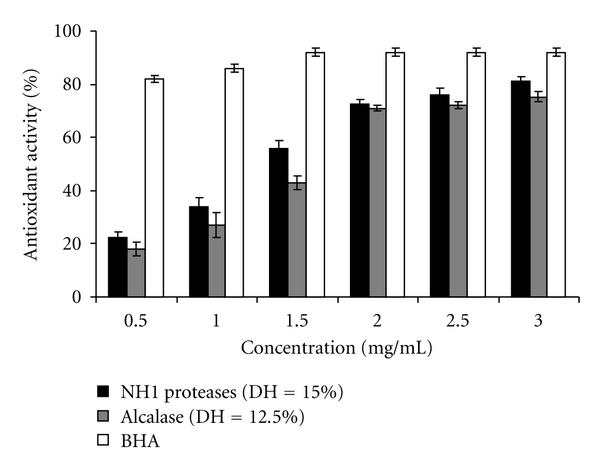
Antioxidant activity using the *β*-carotene bleaching method of NH1-CPH (DH = 15.0%) and Alcalase-CPH (DH = 12.5%) at different concentrations. BHA was used as positive control. Bars represent standard deviations from triplicate determinations.

The antioxidant activity of NH1-CPH (DH = 15%) and Alcalase-CPH (DH = 12.5%) at different concentrations was also evaluated (Figure 6). As can be seen in Figure 6, the antioxidant activity of both cuttlefish protein hydrolysates increased with increasing sample concentration. The NH1 protein hydrolysate showed the highest ability to prevent bleaching of *β*-carotene with 81.11% inhibition at 3 mg/mL. Thereafter, the lower *β*-carotene bleaching in the systems containing these hydrolysates was observed, compared with the system added with BHA. The ability of hydrolysates to prevent the bleaching of *β*-carotene was more likely governed by their amphiphilic properties of amino acids compositions. Localization/orientation at the interface of hydrophobic part of peptides to oil phase and of hydrophilic portion to aqueous phase at the interface is the major principle for emulsion stabilization [60]. The result suggested that protein hydrolysates tested contained peptides with both hydrophilic and hydrophobic portions, in which the peptide segments with hydrophobicity in nature could adsorb at the oil droplet, while the hydrophilic domains preferably suspended in the aqueous phase. The localization of peptides with antioxidative activity at the oil-water interface could favor their antioxidative activity for oil droplet. As a result, the antioxidative activity could be maximized at the interface of emulsion.

### 3.5. Amino Acid Composition

Amino acid compositions of CPHs are shown in Table 6. Both hydrolysates contained histidine and arginine as the major amino acids and were also rich in glutamic acid/glutamine, leucine, lysine, and serine. However, both hydrolysates contained low level of cysteine and proline. From the results, both hydrolysates contained a low level of proline up to 0.29% and 0.33%. 

Based on total amino acids, essential amino acids made up 48.85% and 50.04% of Alcalase-CPH and NH1-CPH, respectively. Therefore, they could serve as the excellent source of useful nutrients. Generally, the differences in amino acid composition between both hydrolysates depended on the existing differences in enzyme specificity and hydrolysis conditions [47]. 

As presented in Table 6, the total content of hydrophobic amino acids of cuttlefish protein hydrolysates obtained at DH of 12.5% and 15% with Alcalase and NH1 proteases was higher, which accounted for 32.36% and 33.64% of the total amino acids, respectively. Amino acids in cuttlefish protein hydrolysates are possibly involved in antioxidative activity. Amino acids have been known to exhibit antioxidant activity; tryptophan and histidine showed high antioxidative activity in comparison with methionine, cysteine, glycine, and alanine [61]. Antioxidative activity of histidine or a histidine containing peptide may be attributed to the chelating and lipid radical-trapping ability of the imidazole ring, whereas the tyrosine residue in the peptide may act as a potent hydrogen donor [16]. Generally, aromatic amino acids are considered to be effective radical scavengers, because they can donate protons easily to electron-deficient radicals. At the same time, their antioxidative stability can remain via resonance structures [62]. From the results, cuttlefish protein hydrolysate had a high nutritional value, based on its amino acid profile.

## 4. Conclusions

The objective of this work was to investigate biochemical properties and the potential antioxidant effect of cuttlefish muscle protein during hydrolysis. The cuttlefish protein hydrolysed with alkaline proteases from *B. licheniformis *NH1 (NH1-CPH) and Alcalase (Alcalase-CPH) resulted products with an excellent solubility over a wide pH range. The antioxidative activity of protein hydrolysate from cuttlefish muscle was governed by enzymes used. Moreover, NH1-CPH and Alcalase-CPH exhibited high antioxidant activity, and the highest activities were obtained with a DH of 15% and 12.5%, respectively. Although both hydrolysates were less effective than positive controls like BHA, fish hydrolysates in general are considered safe products, and they are not subjected to restricted use in foods. Therefore, cuttlefish muscle protein hydrolysate can be used in food systems as a natural additive possessing antioxidative properties.

Further works should be done to isolate and identify the specific peptides in cuttlefish protein hydrolysates that are responsible for the overall antioxidative capability.

## Figures and Tables

**Table 1:** Proximate composition (%) of undigested cuttlefish muscle protein and freeze-dried CPHs^a^.

Compositions (%)	UCMP	NH1-CPH	Alcalase-CPH
Moisture	5.76 ± 0.01	4.13 ± 0.02	4.96 ± 0.04
Protein^b^	79.15 ± 0.48	80.11 ± 0.75	75.36 ± 0.68
Lipids^b^	5.19 ± 0.24	0.68 ± 0.02	0.91 ± 0.05
Ash^b^	6.08 ± 0.71	12.44 ± 0.17	10.12 ± 0.53

^a^Mean ± SD from triplicate determinations.

^b^Dry weight basis.

UCMP: undigested cuttlefish muscle protein.

**Table 2:** Inhibition of lipid peroxidation by CPHs at different degrees of hydrolysis was determined as described in the text after 8 days^a^.

NH1-CPH		Alcalase-CPH	
DH (%)	Inhibition of lipid peroxidation (%)	DH (%)	Inhibition of lipid peroxidation
UCMP	2.00 ± 0.01	UCMP	2.00 ± 0.01
3.6	18.60 ± 1.27	5.6	25.30 ± 2.40
5.5	29.00 ± 1.55	7.6	37.00 ± 2.75
7.2	45.00 ± 1.83	8.7	42.00 ± 1.97
9.1	52.00 ± 2.05	9.6	46.50 ± 2.61
11.1	58.00 ± 2.68	10.8	52.00 ± 2.19
12.9	65.00 ± 1.62	11.6	56.50 ± 2.05
14.5	69.00 ± 1.55	12.3	58.00 ± 1.83
15.0	74.00 ± 1.62	12.5	58.30 ± 2.26

^a^Mean ± SD from triplicate determinations.

**Table 3:** DPPH radical scavenging activity of CPHs with different DH at a sample concentration of 2.0 mg/mL^a^.

NH1-CPH		Alcalase-CPH	
DH (%)	DPPH scavenging activity (%)	DH (%)	DPPH scavenging activity (%)
UCMP	1.67 ± 0.01	UCMP	1.67 ± 0.01
3.6	25.00 ± 1.32	5.6	13.00 ± 1.09
5.5	40.22 ± 1.08	7.6	28.66 ± 1.64
7.2	55.60 ± 1.30	8.7	44.40 ± 1.20
9.1	60.50 ± 1.45	9.6	53.00 ± 1.32
11.1	67.30 ± 1.54	10.8	58.30 ± 0.98
12.9	70.22 ± 1.25	11.6	63.30 ± 1.24
14.5	70.33 ± 2.26	12.3	65.80 ± 1.82
15.0	71.50 ± 0.01	12.5	66.06 ± 1.42

^a^Mean ± SD from triplicate determinations.

**Table 4:** Metal chelating activity of CPHs with different DH at a sample concentration of 2.0 mg/mL^a^.

NH1-CPH		Alcalase-CPH	
DH (%)	Metal chelating activity (%)	DH (%)	Metal chelating activity (%)
UCMP	1.22 ± 0.02	UCMP	1.22 ± 0.02
3.6	26.00 ± 1.32	5.6	16.00± 1.25
5.5	36.00 ± 2.36	7.6	21.00 ± 1.17
7.2	44.60 ± 1.14	8.7	27.00 ± 0.81
9.1	48.80 ± 0.21	9.6	37.00 ± 1.48
11.1	56.40 ± 1.06	10.8	46.40 ± 1.16
12.9	62.00 ± 1.13	11.6	52.00 ± 1.00
14.5	64.00 ± 0.84	12.3	54.00 ± 0.65
15.0	65.00 ± 1.88	12.5	55.60 ± 0.33

^a^Mean ± SD from triplicate determinations.

**Table 5:** Antioxidant activity using the *β*-carotene bleaching method of CPHs with different DH at a sample concentration of 2.0 mg/mL^a^.

NH1-CPH		Alcalase-CPH	
DH (%)	Antioxidant activity (%)	DH (%)	Antioxidant activity (%)
UCMP	1.43 ± 0.01	UCMP	1.43 ± 0.01
3.6	16.50 ± 2.65	5.6	26.50 ± 1.22
5.5	31.50 ± 1.61	7.6	41.50 ± 1.47
7.2	44.50 ± 1.30	8.7	54.50 ± 1.60
9.1	56.50 ± 2.31	9.6	61.50 ± 1.66
11.1	67.50 ± 1.68	10.8	64.50 ± 1.47
12.9	72.00 ± 1.42	11.6	67.50 ± 1.68
14.5	72.20 ± 1.01	12.3	69.50 ± 1.10
15.0	72.60 ± 0.65	12.5	70.90 ± 0.98

^a^Mean ± SD from triplicate determinations.

**Table 6:** Amino acid composition (% mole) of CPHs using Alcalase and NH1 proteases. Values shown are mean values of three measurements.

Amino acid^a^	Alcalase-CPH	NH1-CPH
Aspartic acid	6.01	6.14
Threonine^c^	2.10	2.66
Serine	9.14	9.80
Glutamic acid	11.33	9.54
Glycine	3.05	3.11
Alanine	3.65	3.60
Valine^c^	6.01	6.89
Methionine^c^	2.95	3.01
Isoleucine^c^	3.62	3.81
Leucine^c^	8.78	8.90
Tyrosine	5.80	5.72
Phenylalanine^c^	4.01	3.99
Histidine^c^	11.61	11.23
Lysine^c^	9.77	9.55
Arginine	11.71	11.57
Cysteine	0.17	0.15
Proline	0.29	0.33
TAA^d^	100	100
THAA^d^	32.36	33.64
TEAA/TAA%	48.85	50.04

^a^The aspartic and glutamic acid contents include, respectively, asparagines and glutamine.

^b^Undigested cuttlefish muscle protein.

^c^Essential amino acids.

^d^TAA *≡* total amino acids; THAA *≡* total hydrophobic amino acids; TEAA *≡* total essential amino acids.

## References

[1] Maillard MN, Soum MH, Boivin P, Berset C (1996). Antioxidant activity of barley and malt: relationship with phenolic content. *Lebensmittel-Wissenschaft und-Technologie*.

[2] Butterfield DA, Castegna A, Pocernich CB, Drake J, Scapagnini G, Calabrese V (2002). Nutritional approaches to combat oxidative stress in Alzheimer’s disease. *Journal of Nutritional Biochemistry*.

[3] Halliwell B, Murcia MA, Chirico S, Aruoma OI (1995). Free radicals and antioxidants in food and in vivo: what they do and how they work. *Critical Reviews in Food Science and Nutrition*.

[4] Ito N, Hirose M, Fukushima S, Tsuda H, Shirai T, Tatematsu M (1986). Studies on antioxidants: their carcinogenic and modifying effects on chemical carcinogenesis. *Food and Chemical Toxicology*.

[5] Zhong S, Ma C, Lin YC, Luo Y (2011). Antioxidant properties of peptide fractions from silver carp (*Hypophthalmichthys molitrix*) processing by-product protein hydrolysates evaluated by electron spin resonance spectrometry. *Food Chemistry*.

[6] Khantaphant S, Benjakul S, Kishimura H (2011). Antioxidative and ACE inhibitory activities of protein hydrolysates from the muscle of brownstripe red snapper prepared using pyloric caeca and commercial proteases. *Process Biochemistry*.

[7] Bougatef A, Nedjar-Arroume N, Manni L (2010). Purification and identification of novel antioxidant peptides from enzymatic hydrolysates of sardinelle (*Sardinella aurita*) by-products proteins. *Food Chemistry*.

[8] Bougatef A, Hajji M, Balti R, Lassoued I, Triki-Ellouz Y, Nasri M (2009). Antioxidant and free radical-scavenging activities of smooth hound (*Mustelus mustelus*) muscle protein hydrolysates obtained by gastrointestinal proteases. *Food Chemistry*.

[9] Qian ZJ, Jung WK, Byun HG, Kim SK (2008). Protective effect of an antioxidative peptide purified from gastrointestinal digests of oyster, *Crassostrea gigas* against free radical induced DNA damage. *Bioresource Technology*.

[10] Klompong V, Benjakul S, Kantachote D, Shahidi F (2007). Antioxidative activity and functional properties of protein hydrolysate of yellow stripe trevally (*Selaroides leptolepis*) as influenced by the degree of hydrolysis and enzyme type. *Food Chemistry*.

[11] Thiansilakul Y, Benjakul S, Shahidi F (2007). Antioxidative activity of protein hydrolysate from round scad muscle using alcalase and flavourzyme. *Journal of Food Biochemistry*.

[12] Jun SY, Park PJ, Jung WK, Kim SK (2004). Purification and characterization of an antioxidative peptide from enzymatic hydrolysate of yellowfin sole (*Limanda aspera*) frame protein. *European Food Research and Technology*.

[13] Sathivel S, Bechtel PJ, Babbitt J (2003). Biochemical and functional properties of herring (*Clupea harengus*) byproduct hydrolysates. *Journal of Food Science*.

[14] Wu HC, Chen HM, Shiau CY (2003). Free amino acids and peptides as related to antioxidant properties in protein hydrolysates of mackerel (*Scomber austriasicus*). *Food Research International*.

[15] Pena-Ramos EA, Xiong YL (2002). Antioxidant activity of soy protein hydrolyzates in a liposomial system. *Journal of Food Science*.

[16] Je JY, Park PJ, Kim SK (2005). Antioxidant activity of a peptide isolated from Alaska pollack (*Theragra chalcogramma*) frame protein hydrolysate. *Food Research International*.

[17] Suetsuna K (2000). Antioxidant peptides from the protease digest of prawn (*Penaeus japonicus*) muscle. *Marine Biotechnology*.

[18] Moure A, Domínguez H, Parajó JC (2006). Antioxidant properties of ultrafiltration-recovered soy protein fractions from industrial effluents and their hydrolysates. *Process Biochemistry*.

[19] Balti R, Nedjar-Arroume N, Adjé EY, Guillochon D, Nasri M (2010). Analysis of novel angiotensin I-converting enzyme inhibitory peptides from enzymatic hydrolysates of cuttlefish (*Sepia officinalis*) muscle proteins. *Journal of Agricultural and Food Chemistry*.

[20] Hadj-Ali NE, Agrebi R, Ghorbel-Frikha B, Sellami-Kamoun A, Kanoun S, Nasri M (2007). Biochemical and molecular characterization of a detergent stable alkaline serine-protease from a newly isolated *Sepia officinalis* NH1. *Enzyme and Microbial Technology*.

[21] Kembhavi AA, Kulkarni A, Pant A (1993). Salt-tolerant and thermostable alkaline protease from Bacillus subtilis NCIM No. 64. *Applied Biochemistry and Biotechnology*.

[22] Adler-Nissen J (1986). *Enzymic Hydrolysis of Food Proteins*.

[23] AOAC (1995). *Official Methods of Analysis*.

[24] Tsumura K, Saito T, Tsuge K, Ashida H, Kugimiya W, Inouye K (2005). Functional properties of soy protein hydrolysates obtained by selective proteolysis. *Lebensmittel-Wissenschaft und-Technologie*.

[25] Gornall AG, Bardawill CJ, David MM (1949). Determination of serum proteins by means of the biuret reaction. *The Journal of Biological Chemistry*.

[26] Osawa T, Namiki M (1985). Natural antioxidants isolated from Eucalyptus leaf waxes. *Journal of Agricultural and Food Chemistry*.

[27] Mitsuda H, Yasumoto K, Iwami K (1996). Antioxidative action of indole compounds during the autoxidation of linoleic acid. *Eiyo to Shokuryo*.

[28] Bersuder P, Hole M, Smith G (1998). Antioxidants from a heated histidine-glucose model system. I: investigation of the antioxidant role of histidine and isolation of antioxidants by high-performance liquid chromatography. *Journal of the American Oil Chemists’ Society*.

[29] Decker EA, Welch B (1990). Role of ferritin as a lipid oxidation catalyst in muscle food. *Journal of Agricultural and Food Chemistry*.

[30] Koleva II, Van Beek TA, Linssen JPH, De Groot A, Evstatieva LN (2002). Screening of plant extracts for antioxidant activity: a comparative study on three testing methods. *Phytochemical Analysis*.

[31] Steel RGD, Torrie JH (1980). *Principle and Procedure of Statistics*.

[32] Van der Ven C, Gruppen H, De Bont DBA, Voragen AGJ (2002). Optimisation of the angiotensin converting enzyme inhibition by whey protein hydrolysates using response surface methodology. *International Dairy Journal*.

[33] Hsu KC (2010). Purification of antioxidative peptides prepared from enzymatic hydrolysates of tuna dark muscle by-product. *Food Chemistry*.

[34] Mutilangi WAM, Panyam D, Kilara A (1995). Hydrolysates from proteolysis of heat-denatured whey proteins. *Journal of Food Science*.

[35] Kong X, Zhou H, Qian H (2007). Enzymatic hydrolysis of wheat gluten by proteases and properties of the resulting hydrolysates. *Food Chemistry*.

[36] Benjakul S, Morrissey MT (1997). Protein hydrolysates from pacific whiting solid wastes. *Journal of Agricultural and Food Chemistry*.

[37] Gbogouri GA, Linder M, Fanni J, Parmentier M (2004). Influence of hydrolysis degree on the functional properties of salmon byproducts hydrolysates. *Journal of Food Science*.

[38] Rebeca BD, Pena-vera MT, Diaz-Castaneda M (1991). Production of fish protein hydrolysates with bacteria proteases; yield and nutritional value. *Journal of Food Science*.

[39] Kristinsson HG, Rasco BA (2000). Biochemical and functional properties of Atlantic salmon (*Salmo salar*) muscle proteins hydrolyzed with various alkaline proteases. *Journal of Agricultural and Food Chemistry*.

[40] Liceaga-Gesualdo AM, Li-Chan ECY (1999). Functional properties of fish protein hydrolysate from herring (*Clupea harengus*). *Journal of Food Science*.

[41] Gildberg A (1994). Enzymic processing of marine raw materials. *Process Biochemistry*.

[42] Spinelli J, Koury B, Miller R (1972). Approaches to the utilisation of fish for the preparation of protein isolates; enzymic modifications of myofibrillar fish proteins. *Journal of Food Science*.

[43] Nilsang S, Lertsiri S, Suphantharika M, Assavanig A (2005). Optimization of enzymatic hydrolysis of fish soluble concentrate by commercial proteases. *Journal of Food Engineering*.

[44] Fennema OR (1996). Amino acids, peptides, and proteins. *Food Chemistry*.

[45] Kinsella JE (1976). Functional properties of proteins in foods: a survey. *Critical Reviews in Food Science and Nutrition*.

[46] Sorgentini DA, Wagner JR (2002). Comparative study of foaming properties of whey and isolate soybean proteins. *Food Research International*.

[47] Chobert JM, Bertrand-Harb C, Nicolas MG (1988). Solubility and emulsifying properties of caseins and whey proteins modified enzymatically by trypsin. *Journal of Agricultural and Food Chemistry*.

[48] Dong S, Zeng M, Wang D, Liu Z, Zhao Y, Yang H (2008). Antioxidant and biochemical properties of protein hydrolysates prepared from Silver carp (*Hypophthalmichthys molitrix*). *Food Chemistry*.

[49] Kong BH, Xiong YL (2006). Antioxidant activity of zein hydrolysates in a liposome system and the possible mode of action. *Journal of Agricultural and Food Chemistry*.

[50] Rajapakse N, Mendis E, Jung WK, Je JY, Kim SK (2005). Purification of a radical scavenging peptide from fermented mussel sauce and its antioxidant properties. *Food Research International*.

[51] Shimada K, Fujikawa K, Yahara K, Nakamura T (1992). Antioxidative properties of xanthan on the autoxidation of soybean oil in cyclodextrin emulsion. *Journal of Agricultural and Food Chemistry*.

[52] Theodore AE, Raghavan S, Kristinsson HG (2008). Antioxidative activity of protein hydrolysates prepared from alkaline-aided channel catfish protein isolates. *Journal of Agricultural and Food Chemistry*.

[53] You L, Zhao M, Cui C, Zhao H, Yang B (2009). Effect of degree of hydrolysis on the antioxidant activity of loach (*Misgurnus anguillicaudatus*) protein hydrolysates. *Innovative Food Science and Emerging Technologies*.

[54] Blois MS (1958). Antioxidant determinations by the use of a stable free radical. *Nature*.

[55] Yomauchi R, Tatsumi Y, Asano M, Kato K, Ueno Y (1988). Effect of metal salts and fructose on the autoxidation of methyl linoleate in emulsions. *Agricultural and Biological Chemistry*.

[56] Hultin HO, Shahidi F, Botta JR (1994). Oxidation of lipids in seafoods. *Seafoods: Chemistry, Processing Technology and Quality*.

[57] Samaranayaka AGP, Li-Chan ECY (2008). Autolysis-assisted production of fish protein hydrolysates with antioxidant properties from Pacific hake (*Merluccius productus*). *Food Chemistry*.

[58] Hmidet N, El Hadj Ali N, Zouari-Fakhfakh N, Haddar A, Nasri M, Sellemi-Kamoun A (2010). Chicken feathers: a complex substrate for the co-production of *α*-amylase and proteases by *B. licheniformis* NH1. *Journal of Industrial Microbiology and Biotechnology*.

[59] Bayram T, Pekmez M, Arda N, Yalçin AS (2008). Antioxidant activity of whey protein fractions isolated by gel exclusion chromatography and protease treatment. *Talanta*.

[60] Binsan W, Benjakul S, Visessanguan W (2008). Composition, antioxidative and oxidative stability of mungoong, a shrimp extract paste, from the cephalothorax of white shrimp. *Journal of Food Lipids*.

[61] Riisom T, Sims RJ, Fioriti JA (1980). Effect of amino acids on the autoxidation of safflower oil in emulsions. *Journal of the American Oil Chemists Society*.

[62] Rajapakse N, Mendis E, Byun HG, Kim SK (2005). Purification and in vitro antioxidative effects of giant squid muscle peptides on free radical-mediated oxidative systems. *Journal of Nutritional Biochemistry*.

